# Essential Oils Modulating Inflammation, Oxidative Stress, Endothelial Dysfunction, and Thrombotic Pathways: Relevance to Thromboinflammation and Translational Perspectives

**DOI:** 10.3390/biom16050654

**Published:** 2026-04-28

**Authors:** Valeriu Mihai But, Mahmoud Elsaafin, Mariana Pacurar, Alexandra Mihaela Stoica, Cristina-Ioana Bica, Annamaria Pallag, Mariana Muresan

**Affiliations:** 1 Department of Medicine-Psycho-Neuroscience and Recovery, Faculty of Medicine and Pharmacy, University of Oradea, 410073 Oradea, Romania; dr.butvaleriu@gmail.com; 2 Department of Orthodontics, Faculty of Dental Medicine, George Emil Palade University of Medicine, Pharmacy, Science, and Technology of Târgu Mureș, 38 Gheorghe Marinescu Street, 540142 Targu Mures, Romaniamariana.pacurar@umfst.ro (M.P.); 3 Department of Odontology and Oral Pathology, Faculty of Dental Medicine, George Emil Palade University of Medicine, Pharmacy, Science, and Technology of Târgu Mureș, 540139 Targu Mures, Romania; alexandra.stoica@umfst.ro; 4 Department of Pedodontics, Faculty of Dental Medicine, George Emil Palade University of Medicine, Pharmacy, Science, and Technology of Târgu Mureș, 38 Gheorghe Marinescu Street, 540142 Targu Mures, Romania; cristina.bica@umfst.ro; 5 Department of Pharmacy, Faculty of Medicine and Pharmacy, University of Oradea, 410028 Oradea, Romania; apallag@uoradea.ro; 6 Department of Preclinical Disciplines, Faculty of Medicine and Pharmacy, University of Oradea, 410068 Oradea, Romania; mmuresan@uoradea.ro

**Keywords:** essential oils, thromboinflammation, platelet activation, endothelial dysfunction, oxidative stress, inflammation, eugenol, linalool, vascular biology

## Abstract

Essential oils (EOs) are complex plant-derived mixtures increasingly investigated for their anti-inflammatory, antioxidant, and vasoprotective properties. Thromboinflammation, a process integrating coagulation, platelet activation, endothelial dysfunction, and inflammatory signaling, plays a central role in vascular pathology; however, the contribution of EOs to this process remains insufficiently characterized. This narrative review aims to synthesize current molecular and experimental evidence regarding the effects of EOs and their major bioactive constituents on pathways converging toward thromboinflammation. A focused PubMed/MEDLINE search, supplemented by manual reference screening, was conducted to identify experimental and translational studies on EOs and selected constituents relevant to inflammatory mediators, oxidative stress, endothelial dysfunction, platelet activation, and thrombotic pathways. Available data from predominantly preclinical experimental models indicate that EOs can exert multi-target effects, including modulation of cytokine production, attenuation of oxidative stress, improvement in endothelial function, and inhibition of platelet aggregation, thereby influencing key components of thromboinflammatory pathways. Despite these promising findings, heterogeneity in chemical composition, limited standardization, uncertain exposure relevance, and the predominance of preclinical data remain important limitations. In conclusion, EOs represent a promising but still largely preclinical class of natural compounds capable of modulating interconnected mechanisms relevant to thromboinflammation; however, further translational and clinical studies are required to validate their therapeutic potential.

## 1. Introduction

Thromboinflammation has emerged as a unifying pathobiological framework describing the reciprocal amplification between inflammatory signaling and thrombotic responses. Under physiological conditions, immunothrombosis contributes to host defense by restricting pathogen dissemination and facilitating the clearance of danger signals. However, when excessive, persistent, or spatially dysregulated, the same interconnected mechanisms promote vascular occlusion, tissue ischemia, and organ injury across a broad range of infectious, cardiovascular, metabolic, and inflammatory disorders [1,2]. This shift in perspective has moved thrombosis beyond a purely hemostatic event toward a multicellular and highly coordinated process in which coagulation, innate immunity, platelet activation, endothelial perturbation, and leukocyte recruitment are mechanistically intertwined [1,2,3].

Within this framework, platelet activation, endothelial dysfunction, and oxidative stress should be viewed as tightly coupled components of thromboinflammatory injury rather than as isolated mechanisms. Activated platelets amplify inflammatory and thrombotic signaling through mediator release and leukocyte interactions, dysfunctional endothelium acquires a proadhesive and procoagulant phenotype, and redox imbalance further intensifies cytokine production, leukocyte recruitment, and thrombogenesis. Together, these processes create a self-reinforcing vascular network that helps explain why multi-target modulation may be more relevant than single-pathway intervention in thromboinflammatory disease [3,4,5,6,7].

Among plant-derived bioactive products, essential oils (EOs) are particularly attractive in this regard. These volatile, lipophilic mixtures are composed predominantly of monoterpenes, sesquiterpenes, and phenylpropanoids, with composition varying according to species, chemotype, environmental conditions, harvesting time, and extraction procedures [8,9,10,11,12]. A substantial preclinical literature has shown that EOs can reduce oxidative stress, attenuate proinflammatory signaling, modulate cytokine networks, and influence vascular and cardiometabolic pathways [8,9,10,11]. Nevertheless, these data have usually been interpreted within separate domains, chronic inflammation, immune modulation, endothelial injury, atherosclerosis, or cardiovascular risk-factor management, rather than within the integrative framework of thromboinflammation. Consequently, the relevance of EOs to the platelet–endothelium–inflammation axis remains mechanistically suggestive but conceptually fragmented [9,10,11].

Importantly, a growing number of studies on individual EO constituents support this connection. 1,8-Cineole inhibits GPVI-dependent platelet activation, thrombus formation under flow, and platelet aggregation, and more recent evidence indicates that these effects involve adenosine A2A receptor-dependent cAMP signaling [13,14]. Eugenol, a phenylpropanoid classically associated with clove oil and present in other aromatic matrices, exerts antiplatelet and antithrombotic effects in human and murine models, with reported actions on cPLA2, thromboxane-related pathways, PKC signaling, and NF-κB-linked mechanisms [15,16]. Likewise, linalool and linalyl acetate, major constituents of multiple EOs, have long been recognized for anti-inflammatory activity, while more recent data suggest that linalool can mitigate inflammatory endothelial damage and that linalyl acetate may modulate oxidative stress, inflammation, and endothelial dysfunction [17,18,19]. Collectively, these findings support the view that EO-derived molecules may not act on a single vascular target, but rather across interconnected pathways that converge toward thromboinflammatory responses.

Despite this promise, the translational development of EOs remains challenging. EO composition is inherently variable, and their volatility, low aqueous solubility, instability, and formulation-dependent pharmacokinetics complicate dose standardization, reproducibility, bioavailability, and clinical extrapolation [12,20]. These limitations likely explain why the field contains abundant mechanistic and experimental observations but relatively limited robust translational or clinical evidence. In this context, the present review synthesizes current data on how EOs and selected constituents modulate inflammation, oxidative stress, endothelial dysfunction, and thrombotic pathways, and discusses how these actions may converge on the broader construct of thromboinflammation. By integrating evidence that is usually considered in isolation, this review aims to provide a coherent mechanistic framework for understanding the vascular relevance of EOs and to highlight priorities for future translational research.

**Figure 1:**
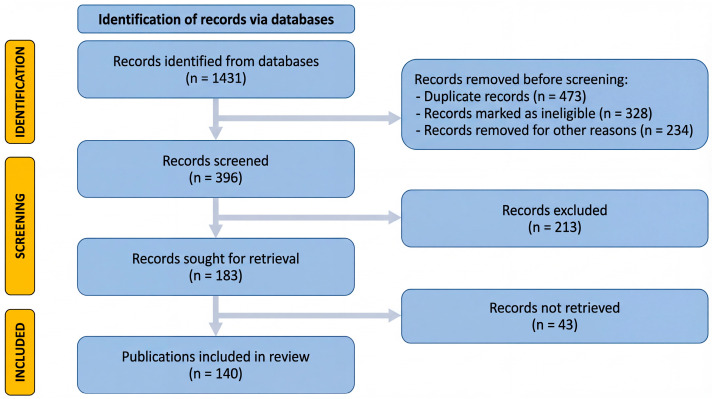
Flow diagram summarizing the scoping search and study identification process that informed this narrative review. The diagram documents literature identification and screening used to guide a mechanism-oriented narrative synthesis and does not represent a formal systematic-review workflow with preregistered eligibility criteria or a dedicated risk-of-bias assessment.

To frame this narrative review, a focused scoping search was conducted in PubMed/MEDLINE up to March 2026 and supplemented by manual screening of the reference lists of relevant articles. Search terms combined descriptors related to EOs and representative bioactive constituents (e.g., “essential oils”, “volatile oils”, “1,8-cineole”, “eugenol”, “linalool”, “linalyl acetate”, “thymol”, “carvacrol”, and “geraniol”) with terms related to thromboinflammation and its component processes (e.g., “thromboinflammation”, “immunothrombosis”, “platelet activation”, “thrombosis”, “endothelial dysfunction”, “oxidative stress”, “inflammation”, “cytokines”, “NETs”, and “coagulation”). A total of 1431 records were initially identified, and the flow diagram shown in Figure 1 summarizes the identification and screening process that informed the present narrative synthesis.

English-language original experimental studies, translational reports, and selected review articles were considered when they provided either direct evidence on thrombotic or thromboinflammatory endpoints or supportive mechanistic relevance to inflammatory signaling, redox regulation, endothelial injury, platelet biology, coagulation, or NET-associated pathways. Priority was given to studies reporting the chemical characterization or dominant constituents of the investigated oils, whereas studies lacking mechanistic relevance or adequate compositional information were deprioritized. Given the narrative aim of this review and the marked heterogeneity of the available evidence, studies discussed in depth were prioritized on the basis of biological relevance, mechanistic depth, and translational value rather than selected solely through algorithmic filtering; older landmark papers were retained when necessary to contextualize more recent findings. Accordingly, Figure 1 documents the scoping search that informed the review and should not be interpreted as a formal systematic-review workflow with preregistered eligibility criteria or a dedicated risk-of-bias assessment.

## 2. Molecular Basis of Thromboinflammation

Thromboinflammation represents a self-amplifying biological network in which inflammation, oxidative stress, endothelial activation, platelet signaling, coagulation, and neutrophil responses are mechanistically intertwined rather than merely coexisting in parallel. In this framework, inflammatory stimuli induce a prothrombotic vascular phenotype, while coagulation proteases, activated platelets, and neutrophil-derived mediators further intensify inflammatory signaling and tissue injury. This reciprocal organization explains why thromboinflammatory disorders frequently involve microvascular obstruction, organ dysfunction, and persistent vascular damage despite conventional antithrombotic therapy, and why they cannot be understood solely through the classical lens of hemostasis [21,22,23,24].

### 2.1. Inflammation

Inflammation constitutes the initiating and perpetuating layer of thromboinflammation. Pathogen-associated molecular patterns (PAMPs) and damage-associated molecular patterns (DAMPs) activate pattern-recognition pathways in monocytes, neutrophils, endothelial cells, and other vascular-resident cells, leading to the release of cytokines and chemokines such as interleukin (IL)-1β, IL-6, tumor necrosis factor-α (TNF-α), and a broader repertoire of leukocyte-recruiting mediators. These inflammatory signals promote leukocyte adhesion, endothelial activation, platelet priming, and local coagulation, thereby lowering the threshold for thrombus formation [21,22,23,24,25,26].

A major mechanistic bridge between inflammation and thrombosis is the induction of tissue factor (TF), particularly in monocytes. TF expression can be driven by Toll-like receptor signaling, inflammatory cytokines, and inflammasome-associated pathways, resulting in activation of the extrinsic coagulation cascade and propagation of thrombin generation. Inflammasome signaling deserves particular emphasis because it not only enhances IL-1 family cytokine maturation but also couples inflammatory activation to pyroptotic membrane remodeling, phosphatidylserine exposure, and release of procoagulant extracellular vesicles. Thus, inflammasome activity does not simply accompany thromboinflammation; it directly reinforces its coagulation arm [24,25,26].

Inflammation also reshapes the vascular microenvironment by enhancing endothelial adhesiveness, favoring platelet–leukocyte aggregate formation, and amplifying DAMP release from injured cells. In this context, inflammation acts as a systems-level coordinator that synchronizes immune sensing, vascular perturbation, and hemostatic activation. Accordingly, thromboinflammation should be viewed as an inflammatory state with hemostatic consequences as much as a thrombotic state with inflammatory consequences [21,22,23,24].

### 2.2. Reactive Oxygen Species and Redox Imbalance

ROS are critical amplifiers of thromboinflammatory signaling. During vascular injury and inflammatory activation, ROS can be generated by multiple sources, including NADPH oxidases, mitochondria, xanthine oxidase, uncoupled endothelial NO synthase, activated platelets, neutrophils, and stressed erythrocytes. Rather than being inert by-products of cellular stress, ROS function as second messengers that intensify inflammatory and prothrombotic pathways [23,27,28,29].

One of the most important vascular consequences of oxidative stress is the reduction in NO bioavailability. This favors vasoconstriction, endothelial dysfunction, platelet adhesion, and leukocyte recruitment. In parallel, oxidative modifications of lipids, proteins, and membrane phospholipids promote redox-sensitive inflammatory signaling, including NF-κB-associated transcriptional responses, further enhancing cytokine release and procoagulant transformation. ROS also contribute to tissue factor induction, phosphatidylserine exposure, endothelial barrier dysfunction, and platelet hyperreactivity [25,27,28,29].

In platelets, ROS potentiate granule secretion, integrin activation, thromboxane-dependent responses, and the development of a procoagulant phenotype. In neutrophils, oxidative burst is tightly linked to NET formation and propagation of sterile inflammatory injury. In endothelial cells, oxidative stress destabilizes intercellular junctions and the glycocalyx, favoring vascular leakage and thromboadhesive transformation. In the evolving thrombus, oxidative processes additionally contribute to fibrin organization, clot persistence, and impaired resolution. Collectively, these observations place ROS at the intersection of inflammation, vascular dysfunction, and thrombogenesis [23,27,28,29,30].

### 2.3. Endothelium

The endothelium is a decisive regulator of thromboinflammatory balance. Under physiological conditions, endothelial cells maintain vascular quiescence by producing NO and prostacyclin, limiting leukocyte adhesion, restraining platelet activation, and expressing anticoagulant regulators such as thrombomodulin, endothelial protein C receptor, and tissue factor pathway inhibitor. The endothelial glycocalyx further reinforces this antithrombotic state by acting as a biophysical and biochemical barrier between circulating blood elements and the vessel wall [27,28,31,32,33].

Upon activation or injury, however, the endothelial phenotype shifts from antithrombotic to proadhesive and procoagulant. Endothelial cells increase the expression of adhesion molecules, facilitate leukocyte tethering and transmigration, and release Weibel–Palade body contents, especially von Willebrand factor (VWF) and P-selectin. This release is central to thromboinflammation because it links endothelial activation to platelet recruitment, microvascular thrombus formation, and inflammatory cell trafficking [27,31,32,34].

The endothelial glycocalyx is particularly important in this transition. Glycocalyx degradation, which occurs in inflammatory and critical illness states, exposes the endothelial surface to circulating cells and alters VWF anchoring and release dynamics. Experimental and conceptual work suggests that the glycocalyx participates in the endothelial handling of VWF, whereas its disruption favors a more prothrombotic surface environment. This is further compounded by inflammation-associated imbalance between VWF and ADAMTS13, which promotes persistence of ultralarge VWF multimers and enhances platelet adhesion under shear conditions [27,28,31,32].

Endothelial cells are also direct targets of extracellular histones and other DAMPs, which can trigger Weibel–Palade body exocytosis and thereby connect sterile inflammation to vascular thrombogenicity. Thus, endothelial dysfunction is not a passive bystander in thromboinflammation; it is a central checkpoint that determines whether inflammatory signaling remains localized and adaptive or becomes disseminated and occlusive [27,28,31,32,34].

### 2.4. Platelets

Platelets are central immune-hemostatic effectors in thromboinflammation. In addition to their canonical role in primary hemostasis, platelets sense vascular injury, inflammatory mediators, immune complexes, and pathogen-related signals, and translate these inputs into adhesive, secretory, and procoagulant responses. This broader role has positioned platelets as dynamic cellular hubs linking coagulation to innate immunity [35,36]. Through receptors such as GPIb-IX-V, GPVI, protease-activated receptors (PARs), Toll-like receptors, FcγRIIA, and integrin αIIbβ3, platelets respond to collagen, VWF, thrombin, NET-associated molecules, and other thromboinflammatory cues. Once activated, they release dense- and α-granule contents, including ADP, serotonin, thromboxane A2, platelet factor 4 (PF4/CXCL4), CCL5/RANTES, and CD40 ligand, all of which amplify leukocyte recruitment, endothelial activation, and thrombus stabilization [35,36,37,38].

A hallmark of platelet participation in thromboinflammation is their ability to form heterotypic aggregates with monocytes and neutrophils. Platelet P-selectin binds PSGL-1 on leukocytes, while additional receptor systems reinforce these interactions and facilitate leukocyte activation. Such contacts can induce monocyte TF expression, stimulate neutrophil activation, and promote NETosis. Furthermore, a subset of highly activated platelets undergoes procoagulant transformation with phosphatidylserine exposure, thereby supporting the assembly of coagulation enzyme complexes and local thrombin generation [25,35,36,37,38,39].

These properties explain why platelet activation contributes not only to clot formation but also to immune amplification, endothelial injury, and propagation of thromboinflammatory damage. In this sense, platelets are not merely effectors downstream of inflammation; they are active organizers of the thromboinflammatory response [35,36,37,38,39].

### 2.5. Coagulation

Coagulation constitutes the enzymatic backbone of thromboinflammation. In most inflammatory settings, coagulation is initiated predominantly through TF-dependent activation of the extrinsic pathway. TF forms a complex with factor VIIa, leading to activation of factors X and IX, amplification of thrombin generation, and formation of cross-linked fibrin. In thromboinflammation, this cascade is not only a hemostatic response to injury but also an immune-adaptive program that can become pathologically exaggerated [25,26,40,41].

Inflammatory monocytes are a major source of inducible TF, and their contribution extends beyond membrane expression to the release of TF-positive extracellular vesicles that disseminate procoagulant activity. Inflammasome signaling and pyroptotic membrane disruption further increase TF activity and phosphatidylserine exposure, providing a mechanistic explanation for the tight coupling between innate immune activation and systemic coagulation [24,25,26].

Thrombin, the central protease of coagulation, is also a potent signaling molecule. Through PAR-mediated effects, thrombin promotes endothelial permeability, platelet activation, leukocyte recruitment, and inflammatory gene expression, thereby reinforcing the very conditions that initiated coagulation. Fibrin itself contributes structurally to the thromboinflammatory microenvironment by providing a scaffold for blood cells and inflammatory mediators. Although TF-dependent initiation predominates in many inflammatory conditions, contact pathway amplification may also become relevant in environments enriched in extracellular DNA and other polyanionic structures, including NETs [26,40,41,42].

At the same time, natural anticoagulant pathways are commonly impaired during thromboinflammation. Reduced thrombomodulin–protein C signaling, diminished antithrombin activity, altered tissue factor pathway inhibitor function, and dysregulated fibrinolytic balance all contribute to thrombin persistence and microvascular clot stability. The result is a feed-forward system in which coagulation proteases amplify inflammation while inflammation suppresses anticoagulant control [23,31,33,40,42].

### 2.6. Neutrophil Extracellular Traps/Immunothrombosis

NETs represent one of the clearest molecular and structural interfaces between innate immunity and thrombosis. NETs are extracellular meshes of DNA decorated with histones, neutrophil elastase, myeloperoxidase, cathepsin G, and additional granular proteins. In physiological host defense, NETs can trap pathogens and spatially confine infection. However, when excessive or insufficiently cleared, they become strong drivers of vascular injury, platelet activation, and thrombus propagation [22,30,37,38,39,43].

NETs contribute to thrombosis through several mechanisms. First, their DNA–protein scaffold physically captures platelets, erythrocytes, and coagulation proteins, thereby stabilizing thrombus architecture. Second, NET-associated histones and proteases directly activate platelets and injure the endothelium. Third, NET-rich environments facilitate activation of coagulation pathways, including contact activation, and may enhance TF-associated procoagulant signaling. Fourth, NET-derived DAMPs further intensify inflammation, producing a powerful feed-forward loop between neutrophils, platelets, and the vessel wall [30,34,37,38,39,43].

Platelets are both inducers and responders within this NET-centered circuit. Activated platelets promote NETosis through adhesive and soluble signaling pathways, whereas NET-associated histones, S100 proteins, and proteases can in turn drive platelet activation and procoagulant platelet formation. Histones also stimulate endothelial Weibel–Palade body exocytosis, linking NETosis to VWF release and endothelial prothrombotic transformation. These interactions are especially relevant in microvascular disease, infection-related coagulopathy, and venous thrombosis [30,34,38,39,43].

In localized and host-protective settings, these responses are commonly referred to as immunothrombosis. Once excessive, prolonged, or spatially uncontrolled, the same molecular machinery evolves into pathological thromboinflammation, characterized by organ injury, microvascular occlusion, and clinically significant thrombotic complications. Thus, NETs do not simply accompany thromboinflammation; they materially organize, stabilize, and propagate it [21,22,23,24,30,34,39,43].

**Figure 2:**
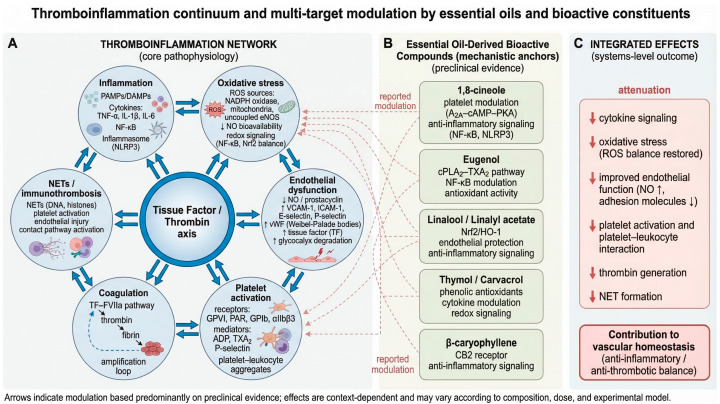
Multi-target modulation of pathways converging on thromboinflammation by essential oils and representative bioactive constituents. (**A**) Core thromboinflammatory network linking inflammation, oxidative stress, endothelial dysfunction, platelet activation, coagulation, and NETs/immunothrombosis around a tissue factor/thrombin-centered axis. (**B**) Representative EO-derived constituents with reported modulatory effects on inflammatory, redox, endothelial, and platelet/thrombotic pathways. (**C**) Integrated downstream effects potentially relevant to thromboinflammation, including attenuation of cytokine signaling, oxidative stress, endothelial activation, platelet–leukocyte interactions, thrombin-generating pathways, and NET-associated amplification. With the exception of limited ex vivo human platelet data and isolated human endothelial observations discussed in the main text, the mechanistic relationships depicted derive predominantly from in vitro and animal studies. Arrows indicate the predominant direction of reported modulation within the cited literature and do not imply comparable effect size, certainty, or clinical relevance across compounds, oils, or experimental models. The figure should therefore be interpreted as a qualitative conceptual synthesis rather than a quantitative ranking of EO activity. Abbreviations: A2A, adenosine A2A receptor; ADP, adenosine diphosphate; CB2, cannabinoid receptor type 2; cAMP, cyclic adenosine monophosphate; cPLA2, cytosolic phospholipase A2; DAMPs, damage-associated molecular patterns; eNOS, endothelial nitric oxide synthase; GPVI, glycoprotein VI; GPIb, glycoprotein Ib; HO-1, heme oxygenase-1; ICAM-1, intercellular adhesion molecule-1; IL, interleukin; NETs, neutrophil extracellular traps; NF-κB, nuclear factor kappa B; NLRP3, NOD-like receptor family pyrin domain containing 3; NO, nitric oxide; Nrf2, nuclear factor erythroid 2-related factor 2; PAMPs, pathogen-associated molecular patterns; PARs, protease-activated receptors; PKA, protein kinase A; ROS, reactive oxygen species; TF, tissue factor; TNF-α, tumor necrosis factor alpha; TREM-1, triggering receptor expressed on myeloid cells 1; TxA2, thromboxane A2; VCAM-1, vascular cell adhesion molecule-1; VWF, von Willebrand factor; αIIbβ3, integrin alpha IIb beta 3.

Taken together, inflammatory signaling, ROS, endothelial dysfunction, platelet activation, coagulation, and NET formation create a highly integrated and self-reinforcing circuit. This mechanistic convergence is particularly important for the present review because it provides the biological rationale for investigating multi-target modulators, such as EOs and their bioactive constituents, that might act simultaneously on several interconnected nodes of the thromboinflammatory network [21,22,23,24,25,26,27,28,29,30,31,33,34,39,42,43]. The core interactions between inflammation, oxidative stress, endothelial dysfunction, platelet activation, coagulation, and NET formation within the thromboinflammatory network are schematically illustrated in Figure 2.

In the present review, “immunothrombosis” is used to denote localized host-protective thrombo-immune responses, whereas “thromboinflammation” refers to the excessive, dysregulated, and tissue-damaging extension of the same machinery.

## 3. Essential Oils and Bioactive Constituents

EOs are complex, volatile, and predominantly lipophilic phytochemical mixtures obtained mainly by steam distillation or hydrodistillation and, in the case of Citrus fruits, by mechanical expression of the epicarp [44,45,46,47]. Rather than representing single natural products, EOs should be viewed as chemically dynamic multicomponent systems. Most oils contain approximately 20–60 constituents, although some may contain substantially more, and in many cases two or three dominant molecules account for a large proportion of the total composition [44,46]. This compositional organization is pharmacologically important because the biological activity of a whole EO cannot always be predicted from its major constituent alone; minor components may modulate the overall effect through additive, synergistic, or antagonistic interactions [44,46,47]. Accordingly, both whole-oil composition and constituent-level analysis are relevant when discussing their vascular and inflammatory actions.

### 3.1. Chemical Definition and Biosynthetic Origin

From a biochemical perspective, EO constituents arise predominantly from two major metabolic lineages: the isoprenoid pathway, which generates terpenes and terpenoids, and the shikimate/phenylpropanoid pathway, which generates aromatic allyl- and propenyl-substituted compounds [45,46,48]. Terpene-derived EO constituents are commonly divided into monoterpenes (C10) and sesquiterpenes (C15), each of which may occur as hydrocarbons or as oxygenated derivatives such as alcohols, aldehydes, ketones, oxides, esters, and phenols [45,46,48]. Phenylpropanoids constitute a chemically distinct yet biologically important group and include molecules such as eugenol, anethole, estragole, and cinnamaldehyde-related compounds [44,45,46,49,50].

This classification is not merely descriptive. The oxidation state, degree of unsaturation, stereochemistry, and functional-group identity of EO constituents influence membrane partitioning, redox behavior, receptor interaction, enzyme modulation, and metabolic fate [45,46,47,48,51]. In other words, subtle chemical differences can have significant pharmacological consequences. For this reason, structurally related molecules found in different EOs may exert overlapping but not identical biological effects, and compounds belonging to different chemical classes may converge functionally on the same biological processes, including inflammation, oxidative stress, endothelial activation, and platelet signaling.

### 3.2. Major Chemical Classes Relevant to the Present Review

Among EO constituents, monoterpenes and oxygenated monoterpenes are especially prominent in the context of vascular and inflammatory biology. Representative examples include 1,8-cineole, linalool, linalyl acetate, limonene, menthol, borneol, geraniol, thymol, carvacrol, and p-cymene-related compounds [17,18,19,45,46,47,48,51,52,53,54]. These molecules are widespread in medicinal and aromatic plants and frequently dominate oils from genera such as Eucalyptus, Lavandula, Mentha, Thymus, Origanum, and Rosmarinus. Their relevance to the present review lies in their repeated association with anti-inflammatory, antioxidant, vasomodulatory, and, in selected cases, antiplatelet effects [13,14,17,18,19,51,52,53,54]. However, prevalence within EOs was not used as the sole criterion for deeper analysis in later sections. Instead, detailed discussion was prioritized for molecules supported either by direct platelet/thrombotic evidence or by recurrent multi-domain effects spanning inflammation, redox signaling, endothelial dysfunction, and thrombosis-related biology. Accordingly, common constituents such as limonene or menthol remain part of the broader EO chemical landscape, but were not selected as primary mechanistic anchors because the currently assembled literature links them less consistently to direct thromboinflammatory endpoints than compounds such as 1,8-cineole or eugenol.

Sesquiterpenes, although often less abundant and less volatile than monoterpenes, can also contribute substantially to EO bioactivity. β-Caryophyllene is particularly noteworthy because it represents a sesquiterpene hydrocarbon with a comparatively well-defined pharmacological profile, including receptor-linked immunomodulatory and anti-inflammatory activity [55,56]. This makes it especially valuable as a mechanistic reference point when discussing EO-derived molecules that act beyond nonspecific membrane interactions.

Phenylpropanoids form another major class of bioactive EO constituents relevant to thromboinflammation-oriented pharmacology. Eugenol is the most important example in the present context because it bridges classical EO pharmacology with pathways directly related to platelet activation, oxidative stress, inflammatory signaling, and thrombosis [15,16,50]. More broadly, phenylpropanoids are characterized by a chemical framework that allows substantial redox and signaling activity, which may help explain their frequent appearance in studies on inflammation and vascular injury [49,50].

### 3.3. Chemotypes, Stereochemistry, and Analytical Standardization

A central concept in EO research is that an oil is not a fixed chemical entity, even when derived from the same nominal plant species. Its composition may vary according to genotype, chemotype, plant organ, phenological stage, geographical origin, climate, altitude, soil characteristics, cultivation conditions, harvesting time, extraction procedure, and storage conditions [12,44,45,46,47]. Consequently, two EOs labeled under the same botanical name may differ markedly in their dominant constituents and, by extension, in their biological activity. This issue is particularly relevant for taxa known to exhibit multiple chemotypes, including *Thymus*, *Origanum*, *Mentha*, and *Lavandula* species [12,46,52].

The chemotype concept has direct mechanistic implications. For example, thyme-type oils may be thymol-rich, carvacrol-rich, linalool-rich, or geraniol-rich, while lavender-derived oils may differ in their relative abundance of linalool, linalyl acetate, camphor, borneol, or 1,8-cineole depending on source and processing [12,45,46,52]. Therefore, pharmacological comparisons across studies are only meaningful when the chemical profile of each investigated batch is reported adequately.

Analytical authentication is equally important. Gas chromatography–mass spectrometry (GC-MS) remains the principal method for EO component identification, while gas chromatography with flame ionization detection (GC-FID) is commonly used for relative quantification. In some cases, enantiomeric analysis is necessary because chirality can influence odor, pharmacokinetics, target binding, and biological response [45,46]. This is highly relevant for compounds such as linalool, menthol, and limonene, whose stereochemical forms are not always biologically equivalent. However, beyond simple constituent listing, batch-specific analytical fingerprinting should be viewed as a prerequisite for meaningful mechanistic interpretation and reproducibility in EO research. Without adequate fingerprinting and transparent reporting of dominant constituents, apparent similarities or discrepancies across studies may reflect compositional divergence rather than true biological consistency or inconsistency.

Inadequate chemical characterization therefore remains one of the major limitations in EO research and a frequent reason for poor reproducibility across experimental studies [12,20,45,46]. In translational terms, insufficient fingerprinting weakens not only inter-study comparability but also confidence that the reported bioactivity of a given EO preparation can be reproduced in subsequent experimental systems or clinical contexts. For this reason, chemotype definition, batch control, and analytical authentication should be considered core methodological requirements rather than optional descriptive refinements.

### 3.4. Whole Oils Versus Isolated Constituents

For the purposes of this review, it is useful to distinguish between whole-oil activity and constituent-driven activity. Whole oils may exert broad, multi-target effects because several constituents act simultaneously on overlapping molecular and cellular pathways. This can be advantageous in complex biological settings such as thromboinflammation, where inflammation, redox imbalance, endothelial dysfunction, platelet activation, and coagulation are tightly interconnected [47,49,51]. At the same time, isolated constituents offer mechanistic clarity by enabling more precise assignment of molecular targets and signaling pathways [49,50,52,53,54,55,56,57].

This distinction should not be interpreted as a competition between reductionist and holistic approaches. Rather, both levels of analysis are complementary. Whole-oil studies provide biologically realistic information about phytochemical mixtures, whereas constituent-centered studies help explain why certain oils repeatedly show activity across experimental systems. In many cases, the pharmacological behavior of an EO is best understood by integrating both perspectives.

### 3.5. Representative Bioactive Constituents Used as Mechanistic Anchors in This Review

Several recurring EO-derived molecules were selected as mechanistic anchors for the present review. This designation was based not on prevalence within EOs alone, but on the availability of comparatively coherent evidence linking a given compound either to direct platelet/thrombotic readouts or to reproducible multi-domain effects across pathways that converge on thromboinflammation. In this sense, the selected anchors were chosen to facilitate mechanistic integration rather than to represent the most abundant EO constituents in general.

First, 1,8-cineole is included because it already has direct experimental evidence for effects on platelet activation, thrombus formation, and hemostatic responses, making it one of the few EO constituents with explicit relevance to thrombotic biology [13,14]. Second, eugenol is included because it represents one of the clearest examples of a phenylpropanoid with convergent anti-inflammatory, antioxidant, antiplatelet, and antithrombotic properties [15,16,50].

Third, linalool and linalyl acetate are included as representative oxygenated monoterpenes with documented anti-inflammatory and redox-modulating properties, as well as emerging relevance to endothelial biology [17,18,19]. Their importance is further reinforced by their prominence in several clinically familiar aromatic oils, particularly those derived from *Lavandula* species. Fourth, thymol and carvacrol are included as phenolic monoterpenes characteristic of thyme- and oregano-type oils, with broad anti-inflammatory, antioxidant, and immunomodulatory profiles [52,53,57]. These compounds are especially useful for illustrating how structurally related monoterpenoid phenols may influence cytokine networks, oxidative injury, membrane-associated inflammatory responses, and, in selected cases, platelet-related pathways.

Finally, β-caryophyllene is included because it broadens the discussion beyond monoterpenes and phenylpropanoids, representing a sesquiterpene with a comparatively more defined receptor-oriented anti-inflammatory profile [55,56]. Its inclusion is conceptually useful because it demonstrates that EO relevance to thromboinflammation is not restricted to a single chemical family.

By contrast, other common EO constituents such as limonene and menthol remain relevant to EO pharmacology and are acknowledged in the broader chemical overview, but were not prioritized as primary mechanistic anchors because, within the literature assembled for the present review, their evidence base is currently less directly connected to platelet biology, thrombosis, or integrated thromboinflammatory endpoints. They should therefore be viewed as important constituents of the wider EO landscape rather than as leading mechanistic reference points for the present synthesis.

Taken together, EOs should be considered chemically heterogeneous but pharmacologically rich systems whose activity reflects both dominant constituents and the broader compositional matrix. The representative compounds highlighted here are not intended to exhaust EO bioactivity, but to provide structurally and mechanistically tractable reference points. In the sections that follow, whole-oil and constituent-level evidence will be integrated to examine how EO chemistry translates into modulation of inflammatory pathways, oxidative stress, endothelial dysfunction, platelet activation, and thrombosis, i.e., the principal processes converging on thromboinflammation [47,48,49,50,51,52,53,54,55,56,57].

## 4. Modulation of Inflammatory and Cytokine Pathways

Inflammatory signaling is one of the clearest domains in which EOs and EO-derived constituents intersect with the biology of thromboinflammation. Across macrophage-, microglia-, and mixed inflammatory-cell models, both whole oils and isolated constituents repeatedly attenuate the production of major pro-inflammatory mediators, especially TNF-α, IL-1β, IL-6, IL-8, MCP-1/CCL2, as well as associated inflammatory effectors such as COX-2, iNOS, NO, and PGE2. This is directly relevant to thromboinflammation because these mediators promote endothelial activation, monocyte recruitment, tissue factor induction, and platelet–leukocyte crosstalk. Importantly, EO anti-inflammatory activity is rarely explained by blockade of a single cytokine; rather, it reflects coordinated modulation of upstream signaling hubs, most consistently NF-κB, MAPK, NLRP3 inflammasome, redox-sensitive signaling, and selected transcriptional regulators linked to immune-cell activation and resolution [21,22,23,24,25,26,31,35,49,50,51,52,53,58,59,60,61,62,63,64,65].

At the level of upstream inflammatory signal transduction, the eucalyptus/1,8-cineole axis provides one of the most mechanistically informative examples. In murine alveolar macrophages, eucalyptus oil and its major constituent 1,8-cineole suppressed LPS-driven inflammatory responses while downregulating the pattern-recognition receptors TREM-1 and NLRP3, together with modulation of NF-κB, p38, JNK/ERK, and the MAPK regulator MKP-1 [58]. This is especially relevant to thromboinflammation because PRR–NF-κB–inflammasome coupling sits upstream of IL-1β maturation, monocyte activation, and procoagulant transformation. The mechanistic scope of 1,8-cineole extends further than cytokine suppression alone: in experimental colitis it downregulated macrophage M1 polarization through the HSP90–NLRP3–SGT1 complex, indicating that this monoterpenoid can influence both inflammatory amplitude and inflammatory-cell phenotype [24,25,26,58,59].

Linalool and eugenol illustrate a second major mechanistic cluster. In RAW 264.7 macrophages and in an LPS-induced lung injury model, linalool reduced TNF-α and IL-6 while blocking the phosphorylation of IκBα, p38, JNK, and ERK, supporting an NF-κB/MAPK-centered mechanism [60]. In BV2 microglia, linalool further reduced TNF-α, IL-1β, NO, and PGE2, while simultaneously suppressing NF-κB and activating the Nrf2/HO-1 pathway, suggesting that inflammatory downregulation and cytoprotective stress responses can be engaged in parallel [61]. Eugenol shows similarly broad cytokine-modulating capacity, but with additional inflammasome relevance: in LPS/ATP-stimulated THP-1 macrophages it reduced IL-1β and IL-6 transcription, COX-2 expression, NF-κB activation, NLRP3 mRNA/protein levels, PANX1 activation, and mature IL-1β release. This profile is particularly interesting for the present review because it connects EO-derived anti-inflammatory activity to the IL-1β/inflammasome axis, a core component of thromboinflammatory amplification [50,60,61,62].

Phenolic monoterpenes and sesquiterpenes add further depth to the cytokine story. In LPS-treated macrophages, carvacrol significantly reduced IL-1β and TNF-α and altered AP-1/NFAT-associated inflammatory transcription programs, while thymol also lowered IL-1β and modulated transcription-factor responses, even if its activity profile was not identical to carvacrol [66]. In a murine sepsis model, carvacrol additionally reduced IL-6 in vivo and in macrophages and acted mainly through ERK1/2 signaling, linking a major EO constituent to a disease context where inflammation and coagulation are tightly coupled [67]. β-Caryophyllene, by contrast, provides an example of receptor-oriented immunomodulation: in inflamed periodontal cells it reduced TNF-α, IL-1β, IL-6, and IL-17A, diminished NF-κB and STAT3 signaling, and restored IL-13, PPARγ, and PGC-1α-associated anti-inflammatory balance via CB2-related mechanisms [68]. Linalyl acetate extends this spectrum even further by modulating TSLP/TSLPR and IL-33 signaling in neuropathic inflammation, suggesting that EO-derived molecules can also influence upstream cytokine circuits beyond the classical TNF-α/IL-1β/IL-6 triad [66,67,68,69].

Whole-oil studies support the idea that cytokine modulation is not an isolated property of a few privileged molecules, but a recurrent pharmacological pattern across chemically diverse oils. Artemisia fukudo essential oil suppressed NO, PGE2, TNF-α, IL-1β, and IL-6 in LPS-activated RAW 264.7 macrophages by inhibiting NF-κB and MAPK activation [65]. Cardamom extracts reduced IL-1β, TNF-α, and IL-8 secretion in LPS-stimulated macrophages, with evidence that NF-κB inhibition contributed to this effect [63]. Likewise, a broader THP-1 screening of Tunisian aromatic and medicinal plant oils identified coriander, geranium, and wormwood EOs as particularly active in lowering IL-6, IL-1β, TNF-α, and COX-2 mRNA expression, underscoring that cytokine attenuation can be reproduced at the level of whole oils even when the dominant constituents differ substantially [63,64,65].

Lavender offers a particularly useful bridge between general EO immunomodulation and the thromboinflammatory focus of this review. External macrophage studies showed that *Lavandula angustifolia* essential oil can markedly inhibit IL-6, IL-8, IL-1β, and TNF-α production in THP-1 cells and can modulate macrophage-mediated inflammatory responses during Staphylococcus aureus challenge [70,71]. In thrombosis-oriented studies, *Lavandula angustifolia* oil in carrageenan-induced thrombosis was associated with lower TNF-α, RANTES, and MCP-1, especially at the higher dose [72]. The same anti-inflammatory direction was maintained in the 2025 streptozotocin-diabetes plus thrombosis model, where lavender oil dose-dependently reduced TNF-α, RANTES, and MCP-1 while contributing to broader inflammatory control [73]. These are not peripheral observations: MCP-1/CCL2 and RANTES/CCL5 are directly relevant to leukocyte recruitment and platelet–leukocyte communication, making this lavender-centered evidence highly compatible with a thromboinflammation-oriented interpretation rather than a purely metabolic one [31,35,36,37,38,70,71,72,73].

Overall, the anti-inflammatory pharmacology of EOs is characterized less by isolated suppression of individual mediators and more by coordinated dampening of cytokine networks, chemokine signaling, inflammasome activation, and inflammatory transcriptional machinery. This multi-target profile is particularly attractive in thromboinflammation, where inflammatory amplification is distributed across several cell types and signaling layers. This section serves as a mechanistic bridge to the subsequent sections on oxidative stress, endothelial dysfunction, and platelet/thrombotic pathways, all of which are tightly integrated with the cytokine networks discussed here [21,22,23,24,25,26,30,31,35,39,43,49,50,51].

## 5. Modulation of Oxidative Stress and Redox Signaling

Oxidative stress is a functional driver of thromboinflammation rather than a passive byproduct of tissue injury. Excessive ROS reduce NO bioavailability, oxidize lipids and proteins, destabilize endothelial homeostasis, enhance platelet responsiveness, and reinforce inflammatory transcriptional programs. In this context, the antioxidant actions of EOs should be interpreted at two complementary levels: direct chemical interception of radical-mediated reactions and indirect regulation of endogenous redox-defense systems. Phenolic EO constituents such as eugenol, thymol, and carvacrol can behave as chain-breaking antioxidants, whereas non-phenolic monoterpenes such as 1,8-cineole, linalool, and linalyl acetate more frequently appear to influence redox balance through signaling-dependent mechanisms involving Nrf2/Keap1, PI3K/AKT, AMPK, Sirt1, mitochondrial preservation, and NOS-related regulation [7,19,29,49,74].

Among EO constituents, 1,8-cineole/eucalyptol provides one of the clearest examples of multilevel redox modulation. In L-NAME-induced hypertension and endothelial injury, 1,8-cineole reduced vascular ROS and MDA, increased SOD and NO availability, improved endothelial structure, and regulated autophagy through PI3K/mTOR signaling [75]. In OGD/R-injured cells, it restored mitochondrial membrane potential, increased SOD, CAT, GSH-Px, HO-1, and NQO1, and reduced ROS, lipid peroxidation, protein carbonyls, MDA, and 8-OHdG via Nrf2 activation [76]. Similar redox-signaling behavior has also been reported in metabolic and toxic injury models: 1,8-cineole directly targeted Sirt1 to enhance Nrf2 signaling, repress oxidative stress, and improve mitochondrial homeostasis in type 2 diabetes [77], while eucalyptol alleviated gentamicin nephrotoxicity with increased GPx and CAT activity, higher Nrf2 expression, and lower oxidative DNA damage [78]. Taken together, these data suggest that cineole-containing oils may influence redox biology not merely by scavenging radicals, but by reprogramming oxidative-stress responses at the signaling level [74,75,76,77,78].

Linalool, linalyl acetate, and lavender-related preparations represent a second relevant redox cluster. Linalool protected against cisplatin-induced nephrotoxicity by restoring antioxidant defenses, attenuating lipid peroxidation, and enhancing Nrf2/HO-1-associated protection while simultaneously suppressing HMGB1/TLR4/NF-κB signaling [79]. In a murine cognitive-impairment model, lavender essential oil and linalool both restored SOD and GPx activity, lowered MDA, and enhanced Nrf2/HO-1 expression, indicating that whole-oil and constituent-level antioxidant actions can converge on the same adaptive pathway [80]. Linalyl acetate further broadened this pattern in diabetic rats exposed to chronic stress, where it restored acetylcholine-induced vasorelaxation, AMPK and eNOS-associated signaling, and serum nitrite levels while improving hemodynamic parameters [81]. Consistent with this broader background, recent lavender oil studies in carrageenan-induced thrombosis and in streptozotocin-diabetes associated with thrombosis showed attenuation of oxidative stress in parallel with anti-inflammatory, antithrombotic, and organ-protective effects, especially at the higher dose [72,73]. These observations are important because they position lavender not as an isolated antioxidant anecdote, but as part of a coherent monoterpene-dominated redox framework with potential thromboinflammatory relevance [19,72,73,79,80,81].

Eugenol and β-caryophyllene extend redox modulation into disease settings with clearer vascular and metabolic implications. In streptozotocin-diabetic rats, eugenol improved endothelium-dependent relaxation and corrected nitrergic deficits, supporting the view that its antioxidant activity has functional vascular consequences rather than purely biochemical ones [82]. In endothelial oxidative-injury models, the eugenol-derived compound aspirin eugenol ester restored Nrf2 signaling, corrected NOS imbalance, improved GSH/GSSG homeostasis, and reduced apoptosis in H_2_O_2_-challenged HUVECs and high-fat-diet vascular injury [83]. More recently, eugenol itself was shown to activate NRF2, HMOX1, and NQO1 in type 1 diabetes, attenuating β-cell oxidative injury and apoptosis [84]. β-Caryophyllene likewise reduced ROS accumulation and NOX2/NOX4 expression under high-glucose conditions [85], attenuated oxidative stress and inflammasome-associated injury in diabetic cardiomyopathy through CB2-dependent AGE/RAGE and PI3K/AKT/Nrf2 signaling [86], and improved pulmonary vascular oxidative status, SOD/CAT activity, and NOS function in monocrotaline-induced pulmonary arterial hypertension, especially when nanoformulated [87]. Together, these findings reinforce the view that EO constituents can modulate redox signaling in a manner that directly intersects with vascular dysfunction, diabetes, and thromboinflammatory predisposition [82,83,84,85,86,87].

Phenolic monoterpenes such as thymol and carvacrol provide an additional mechanistic link between redox chemistry and adaptive antioxidant signaling. In immobilization-stress injury, thymol and p-cymene reduced MDA and inflammatory cytokines, increased glutathione-related defenses, and enhanced Nrf2/HO-1 expression [88]. Carvacrol has likewise been shown to mitigate oxidative injury while engaging Nrf2/HO-1-linked antioxidant responses and dampening NLRP3-associated signaling [89]. Although many of these studies arise from nonvascular models, their mechanistic convergence is striking: EO-derived molecules repeatedly reduce lipid peroxidation and ROS burden, preserve antioxidant enzyme systems, improve NO-related homeostasis, restrain NOX-dependent oxidative amplification, and activate cytoprotective pathways centered on Nrf2, HO-1, NQO1, AMPK, PI3K/AKT, or Sirt1 [74,75,76,77,78,79,80,81,82,83,84,85,86,87,88,89].

From the perspective of thromboinflammation, these redox effects are highly relevant. Oxidative stress lowers endothelial NO bioavailability, facilitates endothelial activation, enhances platelet responsiveness, promotes tissue factor induction, and supports inflammatory amplification [7,21,22,23,24,25,27,29,31,35]. Therefore, the capacity of EOs and their constituents to rebalance ROS generation and antioxidant defenses may represent a core mechanism underlying their broader vascular and thromboinflammatory effects. This does not imply that all EOs act through the same antioxidant logic, nor that direct radical scavenging is sufficient to explain their activity. Rather, the available evidence suggests a spectrum of redox actions ranging from chemical radical-trapping behavior to coordinated modulation of stress-responsive signaling networks. This distinction is especially important for interpreting the later sections on endothelial dysfunction and platelet/thrombotic pathways, where oxidative signaling frequently operates as an upstream determinant rather than a secondary epiphenomenon [7,29,74,75,76,77,78,79,80,81,82,83,84,85,86,87,88,89].

## 6. Essential Oils and Endothelial Dysfunction

Endothelial dysfunction is a pivotal checkpoint in thromboinflammation because it transforms the vascular lining from a quiescent antithrombotic interface into a proadhesive, proinflammatory, and procoagulant surface. Reduced NO bioavailability, impaired eNOS signaling, increased endothelin-1, upregulation of adhesion molecules such as VCAM-1, ICAM-1, E-selectin, and P-selectin, as well as enhanced monocyte adhesion and endothelial apoptosis, all facilitate platelet recruitment, leukocyte trafficking, and local coagulation. In this setting, EOs and EO-derived constituents are particularly attractive because they do not appear to act on a single endothelial abnormality, but rather modulate several determinants of endothelial dysfunction simultaneously, including oxidative stress, inflammatory activation, endothelial vasomotor imbalance, and cellular injury [4,5,6,27,28,29,31,32,90].

One of the best-developed mechanistic clusters involves 1,8-cineole and citronellal. In lipopolysaccharide-induced vascular endothelium dysfunction, 1,8-cineole attenuated endothelial injury through PPAR-γ-dependent regulation of NF-κB, reducing IL-6, IL-8, VCAM-1, and E-selectin in HUVECs while improving vascular endothelial injury in vivo [91]. In a distinct vascular model, 1,8-cineole also ameliorated L-NAME-induced endothelial injury and hypertension through autophagy-related PI3K/mTOR signaling, with improvement in endothelial structure and NO-related balance [75]. Citronellal has shown a similarly coherent endothelial-protective profile: it prevented endothelial dysfunction and reduced atherosclerotic plaque burden in rats [92], attenuated oxidative stress-induced mitochondrial damage and endothelial dysfunction in type 2 diabetes through TRPM2/NHE1-related signaling [93], and more recently alleviated vascular endothelial dysfunction via AP-2α/circRNA_102979/miR-133a-associated mechanisms [94]. Together, these data suggest that selected oxygenated monoterpenes can preserve endothelial function through both classical inflammatory signaling pathways and more specific endothelial homeostatic regulators [75,91,92,93,94].

EOs also influence the endothelial adhesive phenotype, which is especially relevant to thromboinflammation because endothelial activation and monocyte recruitment are early drivers of vascular inflammatory amplification. In HUVECs exposed to high glucose, the essential oil from Fructus Alpiniae zerumbet reduced cell injury, TNF-α, IL-8, ICAM-1, VCAM-1, and NF-κB p65 nuclear translocation [95]. In endothelial cells isolated from umbilical cords of females with gestational diabetes mellitus, anise and laurel EOs significantly reduced monocyte adhesion, VCAM-1 expression, and NF-κB p65 nuclear translocation [96]. A closely related pattern was reported for lavender EO and its main constituents: lavender oil and linalyl acetate suppressed TNF-α-induced E-selectin, P-selectin, VCAM-1, ICAM-1, and phosphorylated NF-κB p65 in endothelial cells, whereas linalool showed a more partial profile [97]. These findings are mechanistically important because they indicate that EO preparations can dampen endothelial activation upstream of leukocyte tethering and platelet–endothelium interactions [21,22,23,24,27,31,35,36,37,38,95,96,97].

The vasomotor component of endothelial dysfunction is also responsive to EO-derived compounds. Linalyl acetate restored acetylcholine-induced vasorelaxation and improved hemodynamic alterations in diabetic rats exposed to chronic immobilization stress, supporting recovery of endothelial NO-dependent function [81]. Eugenol improved vascular dysfunction in streptozotocin-diabetic rats [82] and directly dilated mesenteric arteries by activating endothelial TRPV4 channels, thereby linking a phenylpropanoid EO constituent to endothelial Ca^2+^-dependent vasodilatory signaling [98]. Linalool likewise induced endothelium-dependent vasorelaxation in mouse aortae through soluble guanylyl cyclase and K+ channels [99], and more recent endothelial-cell data showed that (R)-(−)-linalool attenuated LPS-induced endothelial damage, reducing IL-6 and MDA while partially restoring endothelial viability and nitrite-related responses [17]. Taken together, these data indicate that EO relevance to endothelium extends beyond biomarker suppression and includes functional recovery of vascular tone regulation [17,81,82,98,99].

A further layer of endothelial protection involves suppression of senescence, apoptosis, and mitochondrial dysfunction. Geraniol has also shown endothelial-protective effects in vivo, improving endothelial function and attenuating NOX-2-derived oxidative stress in high-fat-diet-fed mice [100]. Whole-oil evidence is also available: the essential oil from the rhizomes of Stahlianthus involucratus improved NO and PGI2, reduced ET-1 and ROS, and attenuated endothelial apoptosis and senescence in ox-LDL-injured HUVECs through Nrf2-mediated preservation of mitochondrial quality [101]. Carvacrol provides an additional example of constituent-level endothelial protection, since in db/db mice and high-glucose endothelial models it alleviated vascular inflammation and endothelial dysfunction while reducing TLR4/NF-κB signaling and proinflammatory cytokines [102]. Collectively, these observations support the view that EO-mediated endothelial benefit is not confined to a single chemical class, but may emerge from monoterpenes, phenylpropanoids, and sesquiterpene-related systems through partially convergent redox-inflammatory mechanisms [100,101,102].

Translational evidence remains limited but is not absent. In a small clinical study, 30 min of inhaled lavender aromatherapy improved brachial artery flow-mediated dilation after night-shift work in healthy medical staff, suggesting that at least some EO preparations may acutely influence endothelial function in humans [103]. However, the field is still dominated by cell and animal models involving LPS, high glucose, ox-LDL, diabetes, and vascular injury paradigms, while differences in chemotype, compositional standardization, dose, and formulation complicate direct comparison across studies [12,20,45,46]. Even so, the available evidence strongly supports endothelial protection as one of the most plausible vascular entry points through which EOs may modulate thromboinflammation. By preserving NO signaling, limiting endothelial adhesion-molecule expression, reducing leukocyte–endothelium interactions, and attenuating endothelial injury, EOs may help restrain the endothelial switch toward a prothrombotic phenotype, thereby setting the stage for their effects on platelet activation and thrombotic pathways discussed in the next section [21,22,23,24,27,31,35,36,37,38,90,91,92,93,94,95,96,97,98,99,100,101,102,103].

## 7. Essential Oils in Platelet Activation and Thrombotic Pathways

Platelet activation is one of the earliest and most functionally decisive events in thromboinflammation, linking vascular injury to thrombus growth, leukocyte recruitment, endothelial activation, and coagulation amplification. In this context, the antithrombotic potential of EOs should not be interpreted solely through classical aggregation assays, but through a broader spectrum of platelet-related endpoints, including granule secretion, integrin activation, clot retraction, platelet–leukocyte aggregate formation, and in vivo thromboembolism models. The currently available literature suggests that selected EOs and EO-derived constituents interfere with several of these nodes simultaneously, often with a particularly strong effect on arachidonic acid/thromboxane-dependent platelet activation, but in some cases also extending to ADP-, collagen-, thrombin-, or immunologically triggered pathways [13,14,15,16,25,26,30,34,35,36,37,38,39,40,41,43,104,105,106,107,108,109,110,111,112,113,114,115].

Among EO constituents, 1,8-cineole is one of the most informative mechanistic examples. Initial work demonstrated that 1,8-cineole inhibits agonist-induced platelet activation, thrombus formation, clot retraction, and haemostatic responses, indicating an effect that extends beyond simple suppression of aggregation [13]. This profile was later mechanistically refined by the demonstration that 1,8-cineole suppresses platelet activation and aggregation through adenosine A2A receptor-dependent activation of the AC–cAMP–PKA pathway, with increased VASP phosphorylation and inhibition of platelet activation markers [14]. More recently, 1,8-cineole was shown to suppress IgG-mediated platelet activation and platelet–leukocyte aggregate formation in an ex vivo SARS-CoV-2 S1-related model, thereby further extending its relevance from thrombosis alone to platelet-mediated inflammatory crosstalk [104].

Phenylpropanoid-rich systems provide equally compelling evidence. Clove oil inhibited human platelet aggregation induced by arachidonic acid, platelet-activating factor, and collagen, reduced thromboxane A2 and 12-HETE production, and protected rabbits against pulmonary platelet thrombosis [108]. Eugenol, the major bioactive constituent of clove oil, showed dual inhibition of platelet-activating factor and arachidonic acid metabolism, suppressed thromboxane-dependent platelet responses, and also protected rabbits from PAF- and AA-induced lethality [109]. Earlier platelet biochemistry studies further showed that eugenol and acetyl eugenol inhibited arachidonate-, adrenaline-, and collagen-induced platelet aggregation, with both compounds outperforming aspirin in selected aggregation settings and acetyl eugenol abolishing AA-induced aggregation at low micromolar concentrations [110,111]. These classical findings are strongly reinforced by recent mechanistic studies showing that eugenol suppresses collagen- and AA-induced platelet activation through inhibition of the PLCγ2–PKC and cPLA2–TxA2 cascade, reduces pulmonary thromboembolism without prolonging bleeding time [15], and additionally modulates a cPLA2–NF-κB axis relevant to platelet plug formation and arterial thrombosis [16].

Whole-oil studies also indicate that antiplatelet and antithrombotic activity is not restricted to clove-derived systems. Foeniculum vulgare EO and its major constituent anethole showed broad inhibition of arachidonic acid-, collagen-, ADP-, and U46619-induced platelet aggregation, prevented thrombin-induced clot retraction, displayed vasorelaxant activity at antiplatelet concentrations, and protected mice against collagen–epinephrine-induced thrombosis without evident prohemorrhagic effects [105]. Likewise, Ocotea quixos EO and its main component trans-cinnamaldehyde inhibited platelet aggregation induced by multiple agonists and reduced thrombin-induced clot retraction; in addition, trans-cinnamaldehyde antagonized thromboxane receptor-mediated vasoconstriction, which is mechanistically relevant to thrombotic vascular responses [106]. Independently, cinnamaldehyde also reduced platelet aggregation and thrombus formation in rodent models, supporting cinnamyl-type EO constituents as bona fide antithrombotic effectors rather than merely vasorelaxant adjuncts [112].

Lavender- and thyme-derived systems are particularly relevant for the logic of this review. Lavender EO inhibited arachidonic acid-, U46619-, collagen-, and ADP-induced platelet aggregation, destabilized thrombin-induced clot retraction, and reduced pulmonary thromboembolism in mice without major prohemorrhagic liability; importantly, its major isolated constituents did not reproduce the full activity of the whole oil, suggesting synergistic multicomponent action [107]. This point is highly relevant when interpreting more recent *Lavandula angustifolia* studies: in carrageenan-induced thrombosis and in streptozotocin-diabetes associated with thrombosis, lavender oil showed antithrombotic benefit in vivo together with anti-inflammatory and antioxidant improvement, even though those studies were not designed primarily as direct platelet-mechanism assays [72,73]. Thyme-derived preparations provide complementary evidence for phenolic monoterpenes. Thymol isolated from Thymus vulgaris inhibited platelet aggregation induced by collagen, ADP, arachidonic acid, and thrombin [113], while a thymol-enriched thyme extract later showed potent inhibition of platelet activation, secretion, and aggregation in human platelets [114]. By contrast, carvacrol appears to exert a milder antiplatelet profile, decreasing thromboxane B2 production and restricting GPIIb/IIIa expression rather than displaying the broader potency reported for thymol- or eugenol-based systems [115].

Taken together, EO-mediated modulation of platelet and thrombotic pathways exhibits several recurring features: agonist-selective inhibition with particular sensitivity of the arachidonic acid/thromboxane axis, interference with platelet secretion and clot retraction, attenuation of P-selectin-dependent platelet inflammatory interactions, and in vivo protection against thromboembolic events in multiple animal models. At the same time, the evidence remains predominantly preclinical and heterogeneous with respect to oil composition, assay platform, and dosing. Even so, the convergence across whole oils and isolated constituents strongly supports platelet activation and early thrombus formation as experimentally supported targets of selected essential oils within the broader thromboinflammatory network [13,14,15,16,72,73,104,105,106,107,108,109,110,111,112,113,114,115]. By contrast, direct evidence that EOs modulate the coagulation arm of this network, including tissue factor induction, thrombin generation, fibrin formation, or fibrinolytic balance, remains comparatively scarce. This imbalance in the current literature suggests that platelet inhibition is substantially better explored than direct coagulation-cascade modulation in EO research. Representative EOs and EO-derived molecules discussed throughout this review are summarized in Table 1, together with their experimental context, principal mechanistic targets, dominant evidence level, and thromboinflammatory relevance.

## 8. Integrative Relevance to Thromboinflammation

By integrating the evidence presented in the preceding sections, the relevance of EOs to thromboinflammation becomes clearer. At the same time, one important distinction must be made explicit: the currently available literature comprises both direct thromboinflammation-relevant evidence and a broader body of supportive mechanistic evidence. Direct evidence is presently concentrated in platelet aggregation and secretion assays, clot-retraction studies, platelet–leukocyte aggregate readouts, and in vivo thromboembolism or thrombosis models. By contrast, a much larger supportive literature derives from inflammation-, redox-, endothelial-, and disease-context models that do not measure thromboinflammation per se, but interrogate pathways that are central to its amplification. The relevance of EOs therefore lies less in the existence of a large body of studies explicitly labeled as “anti-thromboinflammatory” and more in the reproducible convergence of their actions on the interconnected domains that define thromboinflammatory injury [21,22,23,24,25,26,27,28,29,30,31,32,33,34,35,36,37,38,39,40,41,42,43,58,59,60,61,62,63,64,65,66,67,68,69,70,71,72,73,74,75,76,77,78,79,80,81,82,83,84,85,86,87,88,89,90,91,92,93,94,95,96,97,98,99,100,101,102,103,104,105,106,107,108,109,110,111,112,113,114,115].

This distinction is particularly important because thromboinflammation is not driven by a single dominant effector, but by feed-forward crosstalk between endothelial activation, platelet signaling, leukocyte recruitment, redox imbalance, and coagulation. Within this framework, a compound or EO preparation capable of attenuating several of these nodes at once may be more relevant than an agent showing a strong effect in only one isolated assay. This is where selected EO constituents show their most convincing convergence. 1,8-Cineole spans inflammatory regulation, endothelial protection, platelet inhibition, and platelet–leukocyte aggregate reduction; eugenol combines antioxidant, endothelial, antiplatelet, and antithrombotic effects; linalool and linalyl acetate bridge inflammation, oxidative stress, vasorelaxation, and endothelial dysfunction; citronellal and geraniol reinforce the endothelial–redox axis; and thyme-, clove-, fennel-, cinnamon-, and lavender-derived preparations extend these observations at the whole-oil level [13,14,15,16,17,18,19,58,59,60,61,62,63,64,65,66,67,68,69,70,71,72,73,74,75,76,77,78,79,80,81,82,83,84,85,86,87,88,89,90,91,92,93,94,95,96,97,98,99,100,101,102,103,104,105,106,107,108,109,110,111,112,113,114,115]. Viewed together, these data support the interpretation of EOs as multi-target modulators of pathways converging on thromboinflammation rather than as narrowly acting anti-inflammatory or antiplatelet agents [44,45,46,47,48,49,50,51,52,53,54,55,56,57,90,91,92,93,94,95,96,97,98,99,100,101,102,103,104,105,106,107,108,109,110,111,112,113,114,115].

A particularly informative proof-of-concept emerges when multiple thromboinflammatory domains are interrogated within the same experimental model. As discussed in Section 4, Section 5 and Section 7, *Lavandula angustifolia* oil in carrageenan-induced thrombosis and in streptozotocin-diabetes combined with thrombosis improved oxidative, inflammatory, and thrombotic readouts within the same biological systems [72,73]. These studies are conceptually stronger than single-endpoint antioxidant or platelet assays because they capture simultaneous improvement in redox imbalance, inflammatory activation, and thrombosis-related outcomes. They therefore provide integrative examples of how an EO may influence several thromboinflammatory determinants at once, even if the measured endpoints remain only partially aligned with the full mechanistic depth of thromboinflammation [72,73].

At the same time, the available evidence remains insufficient to claim that EOs directly suppress thromboinflammation in the strictest mechanistic sense. Most studies still stop at compartment-level endpoints and do not assess core integrative markers such as tissue factor induction, thrombin-generation signatures, fibrin-formation dynamics, fibrinolytic balance, platelet–neutrophil or platelet–monocyte aggregates, NETosis markers (e.g., citrullinated histone H3, MPO–DNA complexes), DAMP burden (HMGB1, histones, mitochondrial DNA), TF-positive or phosphatidylserine-rich extracellular vesicles, endothelial glycocalyx shedding, or VWF/ADAMTS13 imbalance. In particular, the coagulation arm of the thromboinflammatory network remains far less explored than platelet inhibition, and direct EO evidence for NET-centered endpoints is even more limited. This gap is especially significant because NETs are presented here as a central mechanistic node linking inflammation, thrombosis, and vascular injury; without direct EO studies on NET-associated readouts, the interpretation of EO effects on immunothrombosis remains largely inferential rather than experimentally demonstrated [24,25,27,28,30,31,32,33,34,39,43,104,116,117,118,119,120,121,122].

For this reason, the field is currently best framed as “EOs modulating pathways converging on thromboinflammation” rather than as a fully established anti-thromboinflammatory pharmacology. Future studies should adopt integrated experimental platforms that simultaneously assess inflammatory mediators, redox markers, endothelial dysfunction, platelet activation, coagulation, NETosis, glycocalyx injury, and extracellular-vesicle phenotypes within the same model, particularly in disease settings where thromboinflammation is central, including diabetes, sepsis, atherosclerosis, ischemia–reperfusion injury, and venous thrombosis [21,22,23,24,72,73,116,117,118,119,120,121,122,123,124,125,126]. Overall, the integrative relevance of EOs to thromboinflammation lies not in an already mature direct literature, but in the reproducible overlap of anti-inflammatory, antioxidant, endothelial-protective, antiplatelet, and antithrombotic effects across multiple experimental systems. This framing is scientifically more accurate than claiming a direct, fully established anti-thromboinflammatory role, while still being strong enough to justify EOs as promising multi-pathway candidates for future thromboinflammation-oriented translational research [116,117,118,119,120,121,122,123,124,125,126].

From a critical evidence-ranking perspective, the current EO literature remains uneven. Direct evidence is strongest for platelet-related endpoints, including aggregation, secretion, clot retraction, platelet–leukocyte interactions, and selected in vivo thrombosis models. By contrast, evidence for endothelial protection, redox modulation, and cytokine regulation is broader but often supportive rather than directly thromboinflammatory, because these studies frequently do not include integrated thrombotic or vascular-injury readouts. Evidence for direct modulation of the coagulation cascade, NETosis, endothelial glycocalyx injury, extracellular-vesicle-mediated thromboinflammation, and clinically relevant biomarker endpoints remains sparse or absent. Therefore, the current evidence should be interpreted as a mechanistic plausibility framework rather than proof of established anti-thromboinflammatory efficacy. This evidence hierarchy and the remaining gaps are summarized in Table 2.

## 9. Translational Perspectives and Limitations

### 9.1. Standardization, Chemotypes, and Analytical Fingerprinting

Despite the broad mechanistic activity summarized above, the translational path of EOs toward thromboinflammation-oriented applications remains constrained by a central pharmaceutical problem: the investigational material is often insufficiently standardized. EO composition is shaped by species, chemotype, plant organ, extraction procedure, and storage conditions, while oxidation during storage may further alter both activity and safety. This becomes especially problematic in human studies, where recent appraisals have highlighted incomplete reporting, inconsistent product verification, limited analytical transparency, and frequent methodological weaknesses such as small sample size or short duration. For this reason, chemically defined, batch-controlled EO preparations should be considered a prerequisite for translation rather than a later refinement [12,45,46,127,128,129,130,131,132].

Importantly, the translational problem is not merely variability in abstract terms, but the absence of reproducible analytical fingerprinting in a substantial portion of the literature. When GC-MS/GC-FID profiles, dominant constituents, enantiomeric composition, or batch-specific authentication are incompletely reported, the biological effects attributed to a nominal EO may not be reliably reproducible in later studies. This limitation is especially relevant in vascular and thromboinflammatory research, where small compositional differences could alter platelet, endothelial, or redox-related effects. In this sense, inadequate fingerprinting should be regarded as a major source of interpretive uncertainty rather than a minor reporting defect [12,20,45,46,127,128].

### 9.2. Exposure Relevance, Bioavailability, and Route-Dependent Translation

A second major limitation is exposure relevance. Because EOs are volatile, lipophilic, and chemically labile, the route of administration strongly influences absorption, tissue distribution, efficacy, and toxicity. Recent reviews emphasize that comparative pharmacokinetic and toxicity studies across inhalation, dermal, oral, and other internal routes remain scarce, and that bioavailability data are still insufficient for rational dose translation. For vascular and thromboinflammatory indications, this is a critical gap, because systemic exposure and reproducible target engagement would be required. Advanced pharmaceutical strategies such as nanoemulsions, nanofibers, hydrogels, liposomal systems, and chitosan-based nanosystems are therefore relevant not merely as formulation upgrades, but as enabling technologies to improve stability, solubility, controlled release, and potentially bioavailability [20,127,133,134].

This issue is particularly important because many of the studies discussed in Section 4, Section 5, Section 6 and Section 7 rely on in vitro concentrations whose physiological achievability is rarely addressed. Available human pharmacokinetic data for selected constituents are informative but still limited. For 1,8-cineole, inhalation studies in humans indicate rapid absorption, with peak plasma concentrations reached within minutes and biphasic elimination, demonstrating that systemic exposure can occur but also highlighting strong route dependence and short-term kinetic behavior [135]. For linalool, a recent human oral pharmacokinetic study reported a mean Cmax of approximately 85.5 ng/mL about one hour after a 100 mg dose, with detectable but still low ng/mL systemic exposure and a half-life of approximately 3.9 h [136]. These data are valuable, yet they do not establish that the concentrations effective in many endothelial, anti-inflammatory, or antiplatelet in vitro studies are routinely achievable in humans through standard oral, inhaled, or dermal EO use. This remains one of the most important translational uncertainties in the field.

A further limitation concerns dose–response comparability across the EO literature. Many in vitro and ex vivo studies report biologically active concentrations in isolated cellular, endothelial, or platelet systems, but these concentrations are rarely compared with plasma or tissue exposures achievable through clinically relevant oral, inhaled, or dermal routes. Moreover, EC50/IC50 values, exposure duration, vehicle or solvent conditions, assay matrices, and cytotoxicity thresholds are reported inconsistently across studies. This makes direct potency comparisons between constituents such as 1,8-cineole, eugenol, linalool, thymol, carvacrol, or β-caryophyllene unreliable. Consequently, the field currently lacks a robust quantitative hierarchy of EO-derived compounds, and most available findings should be interpreted as mechanistically informative but not yet dose-validated for clinical translation [20,127,133,134,135,136].

Accordingly, the interpretation of in vitro potency should be cautious. A compound may show clear biological effects in cell or ex vivo systems while still having uncertain exposure relevance in vivo, especially when tested at concentrations that have not been linked to realistic human plasma or tissue levels. This does not negate the mechanistic significance of the findings, but it does mean that much of the current EO literature should be viewed as hypothesis-generating rather than exposure-validated from a clinical pharmacology standpoint [20,127,133,134,135,136].

### 9.3. Safety, Toxicology, and Regulatory Considerations

Safety assessment must likewise go beyond the generic assumption that EOs are inherently safe because they are natural. Existing reviews indicate that toxicity may depend on dose, route, oxidation products, residual contaminants, and patient susceptibility, while skin sensitization, mucosal irritation, and possible interactions in medicated individuals remain important concerns. Oxidation-related sensitization is particularly relevant for stored EO products and is well illustrated by the contact-allergy literature on tea tree oil. In addition, dose–response relationships and safety margins remain insufficiently characterized for many EO constituents, and some compounds may exert cytotoxic or even pro-oxidant effects outside specific concentration windows. For thromboinflammatory applications, this issue becomes especially important because EO candidates with antiplatelet or antithrombotic activity could eventually be combined with conventional antithrombotic therapies. Formal interaction studies and dedicated bleeding-liability assessment should therefore precede any vascular clinical translation [13,14,15,16,104,105,106,107,108,109,110,111,112,113,114,115,127,131,132,133].

Regulatory relevance should also be considered. Product quality, chemical authentication, stability during storage, and transparent reporting of formulation and route are not merely technical details but determinants of whether an EO-based intervention can be meaningfully evaluated, compared, or regulated. In this sense, progress in EO therapeutics will depend not only on pharmacology, but also on adopting standards of product definition and reporting that are closer to those expected for pharmaceutical development [127,128,129].

### 9.4. Human Evidence and Realistic Translational Pathways

Current human EO research is only partially aligned with the requirements of thromboinflammation. Most clinical studies focus on aromatherapy or symptom-oriented outcomes such as anxiety, sleep, pain, and blood pressure, rather than platelet function, endothelial biomarkers, coagulation, NETosis, or extracellular vesicles. Even in cardiovascular populations, a recent meta-analysis in acute coronary syndrome mainly found improvements in anxiety and blood pressure, not a thromboinflammation-specific biological readout. At the same time, the development of Silexan demonstrates that a chemically standardized EO-derived product can reach a drug-like level of clinical evidence when formulation, dose, and indication are tightly defined, although the validated indication is psychiatric rather than vascular [103,129,130,137,138].

For thromboinflammation specifically, the most rational translational route may be staged and biomarker-driven. Future studies should use chemotype-defined, analytically authenticated oils; standardized dosing and route-specific pharmacokinetic assessment; and integrated endpoint panels spanning inflammatory mediators, redox markers, endothelial dysfunction, platelet activation, coagulation, and, where feasible, NET- and extracellular-vesicle-associated readouts. Disease-enriched models such as diabetes plus thrombosis, sepsis, or ischemia–reperfusion should be prioritized over isolated antioxidant assays. Combination strategies with standard therapy are also worth exploring; the adjuvant nadroparin–lavender study is a useful proof-of-concept that EO-based modulation may be more realistic as an adjunct than as a stand-alone antithrombotic approach, although this remains to be tested rigorously [24,25,27,28,30,31,33,34,39,43,72,73,127,128,133,134].

Overall, the translational question is no longer whether EOs can influence pathways relevant to thromboinflammation—they clearly can in preclinical systems—but whether chemically standardized EO-based interventions can deliver reproducible target engagement, acceptable safety, and clinically meaningful benefit in biomarker-driven human studies. Achieving that goal will require a shift from exploratory phytopharmacology toward pharmaceutical standardization, exposure-aware interpretation, rigorous reporting, and indication-focused trial design [127,128,129,130,131,132,133,134,135,136,137,138,139,140,141,142].

## 10. Future Directions

Future studies should move beyond compartmentalized readouts and adopt integrated experimental platforms capable of capturing thromboinflammation as a systems-level process. In practical terms, this means evaluating EO interventions not only through isolated antioxidant or platelet assays, but also through coordinated panels that include inflammatory mediators, redox markers, endothelial activation, platelet–leukocyte interactions, coagulation signatures, NET-associated endpoints, extracellular vesicles, and, where feasible, transcriptomic or other high-dimensional biomarkers. Particularly promising directions include platelet-centered molecular signatures of thromboinflammation and endothelial- or platelet-derived extracellular-vesicle profiling, which may help define biologically responsive patient subsets and improve mechanistic interpretation [24,27,28,29,30,31,32,33,34,39,42,43,118,119,120,139,140]. Future experimental reports should also include complete concentration–response relationships, exposure duration, vehicle and solvent conditions, cytotoxicity thresholds, and, where possible, pharmacokinetic/pharmacodynamic links between in vitro effective concentrations and in vivo systemic or tissue exposure.

From a translational perspective, progress will depend less on identifying additional EO activities and more on improving pharmaceutical rigor. Chemotype-defined and analytically authenticated formulations, route-specific pharmacokinetic characterization, safety and bleeding-liability assessment, and delivery strategies that improve stability and bioavailability should become standard requirements. In parallel, EO-based candidates should be evaluated in disease-enriched models with genuine thromboinflammatory burden, such as diabetes-associated thrombosis, sepsis, atherosclerotic vascular inflammation, ischemia–reperfusion injury, or venous thromboembolism. In analogy with current antithrombotic research, the most realistic therapeutic horizon may not be stand-alone replacement of conventional therapy, but adjunctive modulation of thrombosis while preserving hemostatic safety [12,20,72,73,127,128,129,130,131,132,133,134,135,136,137,138,139,140,141,142].

## 11. Conclusions

EOs and their bioactive constituents emerge from the present review as mechanistically coherent natural products capable of modulating multiple processes that converge on thromboinflammation. Across predominantly preclinical models, the most informative EO-derived systems attenuate inflammatory signaling, rebalance oxidative stress, improve endothelial function, reduce platelet activation, and limit thrombotic propagation. At present, the most defensible conclusion is not that EOs are established anti-thromboinflammatory therapeutics, but that they represent pharmacologically plausible multi-target candidates for thromboinflammation-oriented research. Their current value lies in pathway convergence and hypothesis generation, whereas their future clinical relevance will depend on rigorous standardization, exposure-aware study design, and integrated translational validation.

## Figures and Tables

**Table 1:** Representative essential oils and EO-derived constituents modulating pathways related to thromboinflammation.

Representative EO/Constituent	Type	Experimental Context(s)	In vivo Route/Exposure Context	Main Pathways/Readouts	Dominant Target Domain(s)	Level of Evidence	Thromboinflammatory Relevance	Key Refs
1,8-Cineole	Constituent (oxygenated monoterpene)	Macrophage inflammation; colitis; endothelial injury; platelet assays; thrombosis models	Mixed; route varies across cited in vivo studies; ex vivo human platelet models also included	TREM-1/NLRP3/NF-κB/MAPK ↓; Nrf2/HO-1/NQO1 ↑; PPAR-γ endothelial protection; A2A–cAMP–PKA platelet inhibition	Inflammation; redox; endothelium; platelets	In vitro + in vivo	Representative multi-domain profile	[13,14,58,59,75,76,77,78,91,104]
Eugenol	Constituent (phenylpropanoid)	Macrophage inflammation; diabetic vascular dysfunction; HUVEC oxidative injury; platelet models	Mixed; vascular/diabetes models and in vivo thrombosis models use different exposure contexts	NF-κB/NLRP3 ↓; Nrf2 ↑; NOS modulation; PLCγ2–PKC inhibition	Inflammation; redox; endothelium; platelets	In vitro + in vivo	Representative preclinical profile	[15,16,50,62,82,83,84,98,108,109,110,111]
Linalool/Linalyl acetate	Constituents	Inflammation; nephrotoxicity; endothelial dysfunction	Mixed; route varies across cited rodent models	NF-κB ↓; Nrf2 ↑; AMPK/eNOS protection	Inflammation; redox; endothelium	In vitro + in vivo	Strong endothelial–redox axis	[17,18,19,60,61,79,80,81,97,99]
Geraniol/Citronellal	Constituents	Ox-LDL injury; atherosclerosis	Predominantly rodent in vivo models; route not fully comparable across cited studies	PI3K/Akt/Nrf2 ↑; oxidative stress ↓	Redox; endothelium	In vitro + in vivo	Endothelial/redox dominant	[92,93,94,100]
β-Caryophyllene	Constituent	Inflammation; hyperglycemia	Predominantly oral/gavage in rodent models; nanoemulsion by gavage in PAH model	CB2 activation; ROS ↓; PI3K/Akt/Nrf2	Inflammation; redox; vasculature	In vitro + in vivo	Indirect thromboinflammatory relevance	[55,56,68,85,86,87]
Thymol/Carvacrol	Constituents	Inflammation; sepsis; platelet studies	Mixed; mainly rodent inflammatory models with limited direct in vivo platelet evidence	NF-κB ↓; Nrf2 ↑; platelet aggregation ↓	Inflammation; redox; platelets	In vitro + limited in vivo	Platelet + inflammation link	[52,53,57,66,67,88,89,102,113,114,115]
Lavender EO	Whole oil	Inflammation; thrombosis; diabetes	Intraperitoneal pretreatment in rat thrombosis models; inhalation in limited human endothelial observation	Cytokines ↓; oxidative stress ↓; platelet aggregation ↓	All domains	In vitro + in vivo	Representative integrative preclinical model	[70,71,72,73,97,107]
Clove oil/eugenol-rich systems	Whole oil/enriched system	Human platelet studies; pulmonary thrombosis models	Ex vivo human platelets; rabbit in vivo challenge models (i.v. thrombogenic triggers)	AA-, PAF-, collagen-induced aggregation ↓; TxA2 ↓; 12-HETE ↓; protection against platelet thrombosis	Platelets; thrombosis	Ex vivo + in vivo	Representative platelet/thrombotic anchor with mechanistic support from eugenol	[108,109,110,111]
Foeniculum vulgare EO/anethole	Whole oil + constituent	Platelet aggregation; clot retraction; murine thrombosis	Oral, subacute treatment in mice	Broad inhibition (AA, ADP, collagen, U46619); clot retraction ↓; antithrombotic effects	Platelets; thrombosis	In vitro + in vivo	Robust antiplatelet/antithrombotic profile; limited multi-domain evidence	[105]
Ocotea quixos EO/trans-cinnamaldehyde	Whole oil + constituent	Platelet aggregation; clot retraction; thrombosis models	Oral, subacute treatment in mice	Multi-agonist platelet inhibition; clot retraction ↓; thromboxane receptor antagonism	Platelets; thrombosis	In vitro + in vivo	Good platelet/thrombotic anchor; narrower mechanistic coverage	[106,112]
Endothelium-active whole oils	Whole oils	High-glucose endothelial injury; monocyte adhesion models	N/A (predominantly in vitro evidence)	TNF-α/IL-8 ↓; ICAM-1/VCAM-1 ↓; NF-κB ↓; leukocyte adhesion ↓	Endothelium; inflammation	In vitro + limited in vivo	Endothelial inflammatory priming and leukocyte recruitment	[95,96]

The table summarizes experimental contexts, principal molecular pathways and readouts, dominant target domains (inflammation, redox balance, endothelium, platelets, and coagulation), level of evidence, and overall thromboinflammatory relevance. Evidence is derived predominantly from in vitro, ex vivo, and in vivo preclinical models, including macrophage and endothelial cell systems, platelet function assays, and experimental thrombosis models. Arrows indicate direction of modulation (↑ increase/activation; ↓ decrease/inhibition). Effects are context-dependent and may vary according to compound composition, dose, and experimental model. The “In vivo route/exposure context” column summarizes representative or predominant administration patterns across the cited literature and is not intended as an exhaustive pharmacokinetic comparison. For rows pooling highly heterogeneous studies, route is summarized broadly rather than study-by-study. Abbreviations: AA, arachidonic acid; ADP, adenosine diphosphate; AMPK, AMP-activated protein kinase; CB2, cannabinoid receptor type 2; cAMP, cyclic adenosine monophosphate; eNOS, endothelial nitric oxide synthase; EO, essential oil; HETE, hydroxyeicosatetraenoic acid; HO-1, heme oxygenase-1; HUVEC, human umbilical vein endothelial cells; ICAM-1, intercellular adhesion molecule-1; IL, interleukin; MAPK, mitogen-activated protein kinase; NF-κB, nuclear factor kappa B; NLRP3, NOD-like receptor family pyrin domain containing 3; NQO1, NAD(P)H quinone dehydrogenase 1; NOS, nitric oxide synthase; Ox-LDL, oxidized low-density lipoprotein; PAF, platelet-activating factor; PI3K, phosphoinositide 3-kinase; PKC, protein kinase C; PKA, protein kinase A; PLCγ2, phospholipase C gamma 2; PPAR-γ, peroxisome proliferator-activated receptor gamma; ROS, reactive oxygen species; TREM-1, triggering receptor expressed on myeloid cells 1; TNF-α, tumor necrosis factor alpha; T_X_A2, thromboxane A2; VCAM-1, vascular cell adhesion molecule-1; 12-HETE, 12-hydroxyeicosatetraenoic acid.

**Table 2:** Direct thromboinflammatory evidence, supportive mechanistic evidence, and major research gaps in current EO research.

Domain/ Integrative Node	Examples of Informative Endpoints	Current EO Evidence Status	Predominant Evidence Level	Principal Gap/Priority for Future Studies
Inflammatory cytokine networks	TNF-α, IL-1β, IL-6, MCP-1/CCL2, RANTES/CCL5, NF-κB, NLRP3	Broad supportive evidence	Mainly in vitro and animal in vivo	Need integration with direct thrombotic endpoints in the same models
Oxidative stress/redox signaling	ROS, MDA, SOD/CAT/GPx, Nrf2/HO-1, NO bioavailability	Broad supportive evidence	In vitro + animal in vivo	Need route-aware exposure interpretation and disease-integrated designs
Endothelial dysfunction	VCAM-1, ICAM-1, E-selectin, NO/eNOS, vasorelaxation, monocyte adhesion	Moderate supportive-to-direct evidence	In vitro + animal in vivo; limited human	Need standardized formulations and biomarker-linked human studies
Platelet activation/early thrombosis	Aggregation, secretion, clot retraction, P-selectin, platelet–leukocyte aggregates, thromboembolism models	Strongest direct evidence	In vitro, ex vivo human, animal in vivo	Need comparative potency, PK relevance, and bleeding-liability assessment
Coagulation cascade	Tissue factor induction, thrombin generation, fibrin formation, fibrinolysis	Scarce direct evidence	Mostly indirect/sparse	Major experimental gap
NETosis/immunothrombosis	citrullinated histone H3, MPO–DNA complexes, extracellular DNA, direct NETosis assays	Minimal to absent direct evidence	Largely absent	Major mechanistic gap requiring integrated neutrophil–platelet–endothelium models
Endothelial glycocalyx/VWF–ADAMTS13 axis	Glycocalyx shedding markers, VWF multimers, ADAMTS13 activity	Essentially absent	Absent/near absent	Key vascular-integrative gap
Extracellular vesicles/DAMP-related readouts	TF-positive EVs, phosphatidylserine-rich EVs, HMGB1, histones, mitochondrial DNA	Essentially absent	Absent/near absent	Important biomarker and mechanism gap
Human translational evidence	PK, route-dependent exposure, target engagement, safety margins, vascular biomarkers	Very limited	Limited human data	Critical translational gap

This table provides a qualitative synthesis of the current literature and is not intended as a formal risk-of-bias assessment or quantitative ranking of EO efficacy. “Direct evidence” refers to studies assessing thrombotic or thromboinflammatory endpoints such as platelet aggregation/secretion, clot retraction, platelet–leukocyte aggregates, or in vivo thrombosis/thromboembolism models. “Supportive mechanistic evidence” refers to studies addressing inflammatory, redox, endothelial, or related disease-context pathways that are biologically relevant to thromboinflammation but do not directly measure integrated thromboinflammatory endpoints. The stated evidence level reflects the predominant type of currently available data. Abbreviations: ADAMTS13, A Disintegrin and Metalloprotease with Thrombospondin motifs 13; EVs, extracellular vesicles; HMGB1, high-mobility group box 1; ICAM-1, intercellular adhesion molecule-1; MPO, myeloperoxidase; NETosis, neutrophil extracellular trap formation; NO, nitric oxide; eNOS, endothelial nitric oxide synthase; ROS, reactive oxygen species; TF, tissue factor; VCAM-1, vascular cell adhesion molecule-1; VWF, von Willebrand factor.

## Data Availability

Data sharing is not applicable to this article as no new data were created or analyzed in this study.
